# A new species of *Licania* (Chrysobalanaceae) from Cordillera del Cóndor, Ecuador

**DOI:** 10.3897/phytokeys.26.4590

**Published:** 2013-09-27

**Authors:** Ghillean T. Prance

**Affiliations:** 1 Royal Botanic Gardens, Kew, Richmond, Surrey, TW9 3AB, UK

**Keywords:** Chrysobalanaceae, *Licania*, Cordillera del Cóndor, Ecuador

## Abstract

A new mid altitude species of the predominantly lowland genus *Licania*, *Licania condoriensis* from Ecuador is described and illustrated.

## Introduction

A worldwide monograph of the Chrysobalanaceae was published in 2003 (Prance and Sothers 2003a, b). Some recent collections from Ecuador made in 2005 are of an undescribed species of *Licania*. This genus of 218 speciesis predominantly a lowland one and all three collections of this new species, *Licania condoriensis*, are from an altitude of over 1,100 m. Table 1 lists 14 montane and submontane species of *Licania* that occur mainly at altitudes of over one thousand metres.

## Description

### Licania condoriensis

Prance sp. nov.

urn:lsid:ipni.org:names:77132009-1

http://species-id.net/wiki/Licania_condoriensis

Fig. 1

Ab *Licania compacta* foliis subcoriaceis, haud nitidibus, haud dense brunneo-tomentosis, staminibus 7-8 differt.

#### Type.

Ecuador. Zamora-Chinchipe, El Pangui, Cordillera del Cóndor, plateau of Contrafuerte, Tres Patines, W of main Cóndor ridge above Jardin Botánico of EcuaCorriente Copper Company, 03°37'48"S, 78°26'50"W, 1685 m. 2 Dec 2005, *D. Neill & W. Quizhpe 15076* (holotype K; isotypes, MO, QCNE).

Small tree 3–7 m tall, young branches appressed puberulous, soon glabrous. Leaf lamina broadly ovate, 2.5–6 × 1.5–3.5 cm, subcoriaceous, acute or with short blunt acumen at apex, rounded to subcuneate at base, margins entire, glabrous and dull with densely reticulate prominulous venation above, with well-developed stomatal cavities beneath, the venation flattened around slit-like apertures of cavities, the venation glabrous and the cavities filled by a mass of white, unicellular simple hairs; veins 13–15 pairs, plane above, prominulous beneath; petioles glabrous, 3–10 mm long, rugose, with two sessile glands near apex. Stipules lanceolate, early caducous. Inflorescences short once-branched panicles 3–5 cm long, lateral branches borne at 90 degrees to rachis, the rachis and branches densely yellowish-brown tomentose. Flowers c 2 mm long, sessile or subsessile on primary branches of inflorescence; bracteoles oblong-triangular, acute, 1–1.5 mm long, tomentose on exterior and with ciliate margins. Receptacle campanulate, 1.5 mm long, yellowish-brown tomentose on exterior; calyx-lobes c. 1 mm long, acute, triangular, densely tomentose on exterior, tomentose within; petals absent; stamens 7–8, included, filaments equaling or shorter than the calyx lobes in length, c. 0.8 mm long. Ovary inserted at base of receptacle, unilocular with 2 ovules; style pubescent at base. Young fruit only seen, puberulous, glabrescent, unilocular.

#### Additional specimens examined.

Ecuador. Morona-Santiago: Limon Indanza, Cordillera del Cóndor, Centro Shuar Yunkumas, Cerro Chuank Naint, 03°03'31"S, 78°14'48"W, 1,130 m, 19 Dec 2005, *A. Wisum & Grupo Shuar de Conservación 326* (K, MO, QCNE); same locality, Asociación Nunkui, 03°3'34"S, 78°14'45"W, 1,150 m, 19 Dec 2005, *C. Morales, A. Wisum & C. Kajekai 1593* (K, MO, QCNE).

This distinct mid-altitude *Licania* belongs to Section *Licania* of the genus and is probably most closely related to *Licania compacta* Fritsch from Roraima in Guyana, but differs from *Licania compacta* in the much less coriaceous, more acute leaves with a dull rather than shiny upper surface and in lacking the dense pubescence that covers the stomatal cavities of the latter and in the greater number of stamens. The leaves of *Licania condoriensis* are very similar to those of *Licania octandra* (Hofmanns. ex Roem & Schultes) Kuntze subsp. *octandra*, but it differs in the fewer stamens (7-8 vs 9-12) that are included rather than far exserted. All collections of *Licania condoriensis* are from the Cordillera del Cóndor for which this species is named.

**Figure 1.**
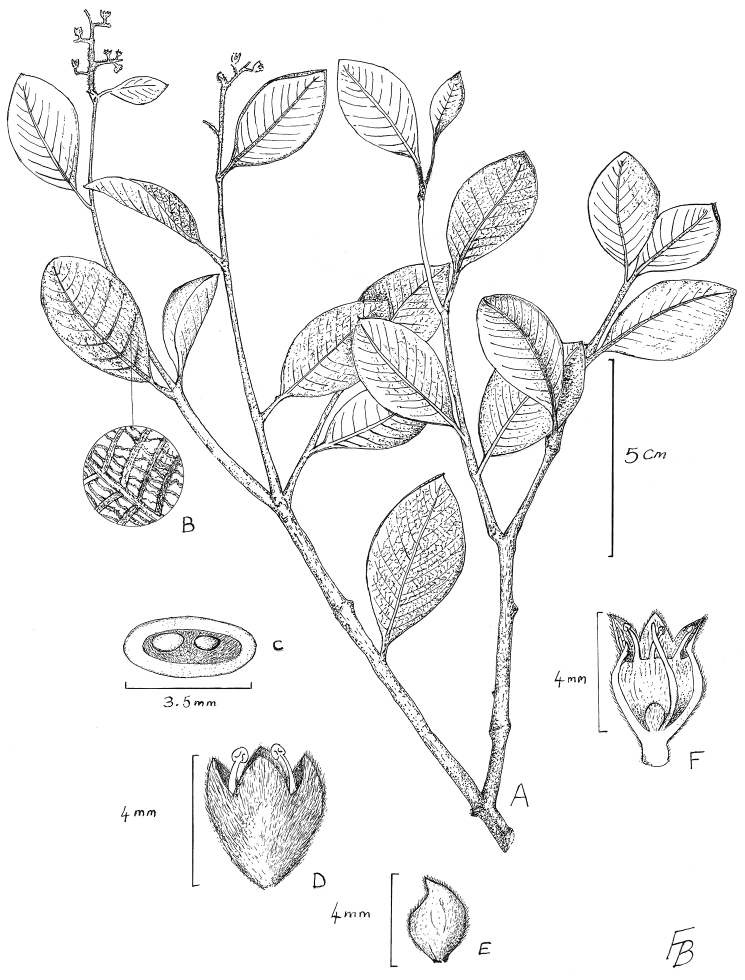
*Licania condoriensis* Prance: **A** habit **B** leaf undersurface showing reticulation from the deep stomatal cavities **C** ovary cross section **D** flower **E** bracteole **F** flower section. (Drawn from *Neill & Quizhpe 15076* by Flora Bamford).

**Table 1.** Species of *Licania* (Chrysobalanaceae) occurring mainly at above 1000 m.<br/>

**Species**	**Locality**	**Altitude**
***Licania* subgenus *Moquilea* section *Moquilea***		
*Licania durifolia* Cuatr.	Colombia, Ecuador, Peru	500–2000 m
*Licania cabrerae* Prance	Colombia: Antioquia	2200–2550 m
*Licania montana* Prance	Venezuela: Lara	1300–1500 m
*Licania hedbergii* Prance	Ecuador: Napo	1600 m
*Licania longicuspidata* Prance	Ecuador: Carchi	650–1800 m
*Licania cariae* A. Cardozo	Venezuela: Aragua	1100–2000 m
*Licania chiriquiensis* Prance	Panama: Chiriqui	1007–1200 m
***Licania* subgenus *Moquilea* section *Leptobalanus***		
*Licania jefensis* Prance	Panama: Chiriqui	1007 m
***Licania* subgenus *Licania* section *Hymenopus***		
*Licania pakaraimensis* Prance	Venezuela: Bolívar	1400 m
***Licania* subgenus *Licania***		
*Licania subrotundata* Maguire	Venezuela: Dist. Federal	1200–2000 m
*Licania aracaensis* Prance	Brazil: Amazonas	1000 m
*Licania pittieri* Prance	Venezuela: Aragua	1100–2200 m
*Licania tepuiensis* Prance	Venezuela: Bolívar	1350 m
*Licania condoriensis* Prance	Ecuador: Zamora-Chinchipe	1130–1685 m

## Supplementary Material

XML Treatment for Licania condoriensis
